# On-Site Fecal Sludge Treatment with the Anaerobic Digestion Pasteurization Latrine

**DOI:** 10.1089/ees.2016.0148

**Published:** 2016-11-01

**Authors:** Aaron A. Forbis-Stokes, Patrick F. O'Meara, Wangare Mugo, Gelas M. Simiyu, Marc A. Deshusses

**Affiliations:** ^1^ Department of Civil and Environmental Engineering, and Duke Global Health Institute, Duke University, Durham, North Carolina.; ^2^ Wataalamu Repair & Maintenance, Eldoret, Kenya.; ^3^ Department of Environmental Biology and Health, University of Eldoret, Eldoret, Kenya.

**Keywords:** anaerobic digestion, biological treatment processes, disinfection, energy use and resources, fecal waste, thermal treatment

## Abstract

The Anaerobic Digestion Pasteurization Latrine (ADPL) is a self-contained and energy neutral on-site sanitation system using anaerobic digestion of fecal sludge to generate biogas and then uses the biogas to pasteurize the digester effluent at 65–75°C to produce a safe effluent that can be reused locally as a fertilizer. Two ADPL systems were installed on residential plots with 17 and 35 residents in a peri-urban area outside of Eldoret, Kenya. Each system comprised three toilets built above a floating dome digester and one heat pasteurization system to sanitize the digested effluent. ADPLs are simple systems, with no moving parts and relying on gravity-induced flows. Adoption at the two sites was successful, and residents reported that the systems had little to no odor or flies. ADPLs were monitored for biogas production and temperatures in the pasteurization system. ADPLs serving 17 and 35 residents produced on average 16 and 11 L_biogas_/person/day (maximum of 20 and 15 L_biogas_/p/d), respectively. The temperature in the sterilization system was greater than 65°C on 58% and 87% of sampling days during the most stable period of operation. Treated effluent was analyzed periodically for chemical oxygen demand (COD), biochemical oxygen demand (BOD), total ammonia nitrogen (TAN), pH, and fecal coliform (FC). On average, the effluent at the two locations contained 4,540 and 6,450 mg COD/L (an 85% or 89% reduction of the estimated input), 2,050 and 3,970 mg BOD/L, and 2,420 and 4,760 mg NH_3_-N, respectively, and greater than 5 log reductions of FC (nondetectable) in the sterilization tank. Results from this field study show that anaerobic digestion of minimally diluted fecal sludge can provide enough energy to pasteurize digester effluent and that the ADPL may be a suitable option for on-site fecal sludge treatment.

## Introduction

The 2015 Millennium Development Goals (MDG) report contains alarming facts as follows: 32% of global population, or about 2.4 billion people, still do not have access to improved sanitation while 946 million practice open defecation (United Nations, 2015a). Global sanitation coverage increased over the MDG period, but fell short of the goal, particularly in developing countries. The sub-Saharan Africa and Oceania regions were the furthest from their goal, each falling 32 percentage points behind target (current coverage at 30% and 35%, respectively). The increase in coverage of improved sanitation of 68% is promising; however, if treatment of sewage before release in the environment was included in the definition of “improved sanitation,” only 40% of the population would have improved sanitation (Baum *et al.*, 2013). A case study of Dhaka, Bangladesh, found that 99% of those living in urban areas had access to improved sanitation with either sewage connections or on-site facilities, but only 1% of the waste was treated before entering the environment, meaning that 99% of total fecal waste in Dhaka ends in the environment untreated and, thus, can contribute to spreading diarrheal diseases (World Bank *et al.*, 2014). In an effort to address the need for waste treatment, the Sustainable Development Goals (SDGs) established Goal 6.3, which includes halving the proportion of untreated wastewater entering the global environment (United Nations, 2015b).

Diarrhea contributes to more global deaths than HIV/AIDS, measles, and malaria combined (World Health Organization, 2015a), and in the sub-Saharan Africa region, in 2012, diarrhea attributed to inadequate sanitation contributed to an estimated 126,000 deaths (Prüss-Ustün *et al.*, 2014). In addition, diarrhea is the second-highest cause of death for children less than 5 years of age. Water- and sanitation-related diarrheal diseases led to the death of 500,000 children in 2013 alone (UNICEF, 2013). Up to 2.5 billion cases of childhood diarrhea associated with fecal contamination are reported each year, and 24% of the global population is affected by soil-transmitted helminth infections from fecal contamination (Bill and Melinda Gates Foundation [BMGF], 2011; World Health Organization, 2015b). These facts stress that continued efforts in provision of improved sanitation and treatment are greatly needed.

The need for nonsewered sanitation is high as 2.7 billion people currently rely on on-site sanitation, and the number is expected to grow to 5 billion by 2030 (Strande, 2014). On-site systems are commonly seen as rural or temporary solutions, but have become increasingly important for urban populations as 1 billion people using on-site systems live in urban areas of Africa, Asia, and Latin America. In sub-Saharan Africa, 65–100% of sanitation in urban areas is through on-site systems (Strande, 2014). On-site sanitation is necessary for growing urban populations as the centralized sewer-based collection and treatment systems existing in developed nations are too costly, too complex, and use too much water and/or energy to implement in poor and less developed countries (Lalander *et al.*, 2013; Mara, 2013). As the global demand for improved water and sanitation increases, treatment technologies that minimize wastes, as well as water consumption, and allow water reuse will be critical to meet the SDGs (Gijzen, 2001; Katukiza *et al.*, 2012).

Anaerobic digestion is the process of biologically breaking down organic wastes in absence of oxygen. It yields two outputs as follows: an effluent that is highly reduced in organic content and biogas—a mixture of methane (55–70% of total volume makeup) and carbon dioxide (30–45% volume) with trace concentrations of hydrogen sulfide (H_2_S), hydrogen, and water vapor. Anaerobic digestion has been shown to have advantages compared to other treatment options because of its low operational costs and positive energy balance, allowing recovery of the energy content and potential reuse of the nutrients present in the original waste stream (Gijzen, 2002; McCarty *et al.*, 2011). The process is robust and efficient, has low sludge production, can withstand high organic loading rates, and has less operational complexity compared to other traditional wastewater treatment methods (Rittmann and McCarty, 2001). Previous studies found an expected biogas production of 20–25 L per person per day (L/p/d) for domestic sewage (Lettinga *et al.*, 1993; Kujawa-Roeleveld *et al.*, 2003; Chaggu *et al.*, 2007; Mang and Li, 2010; Tilmans *et al.*, 2014) and 28–35 L/p/d for fecal sludge (Kujawa-Roeleveld *et al.*, 2006; Lohri *et al.*, 2010), corresponding to 120–210 Wh/p/d of energy recovered.

Although efficient for organic matter conversion, anaerobic digestion alone does not provide sufficient treatment for most applications. Removal of fecal indicators in anaerobic reactors is low with typically only one log reduction of fecal coliform (FC) (Chernicharo *et al.*, 2001; von Sperling et al., 2002, 2004; Sahlström, 2003; von Sperling and Mascarenhas, 2004), there is minimal effect on inactivating *Ascaris suum* at mesophilic temperatures (Popat *et al.*, 2010; Manser *et al.*, 2015), and little to no nutrient (N, P) removal is achieved. Conventional disposal of effluent from on-site systems using leach beds and soil absorption is unsafe in regions with environmental constraints (e.g., rocky soils, high water table, and heavy seasonal rain) and results in the spread in soil-transmitted helminth diseases. SDG 6.3 aims to promote the increase of global waste reuse; however, where reuse of treated sewage (e.g., for land application as a fertilizer) is desired, pathogen inactivation is crucial for safe handling and reuse (United Nations, 2015b); in the case of anaerobic digestion, pathogen-free effluent can only been guaranteed by additional treatment. One alternative method for pathogen reduction is pasteurization, the process of time and temperature exposure for inactivation of pathogens. Based on the study by Feachem *et al.* (1981), a time of 10–15 min at 70°C (highest temperature tested) or 50 min at 65°C is required to be in the “safety zone” for full inactivation of the pathogens selected for the study, which included *Vibrio cholerae*, shigella, and ascaris eggs.

In light of the shortcomings of anaerobic digestion and advantages of pasteurization, the overall objective of this work was to combine the aforementioned technologies, using biogas produced by anaerobic digestion of mostly undiluted human fecal wastes to power its own pasteurization system to sanitize the digester effluent. The aim was to yield a self-contained and energy neutral sanitation system that could treat organics and eliminate pathogens from fecal waste. This system was called and will hereon be referred to as the Anaerobic Digestion Pasteurization Latrine (ADPL). The concept was tested in the laboratory with simulant feces and urine for over 1 year with a 17 L floating dome digester (Colón *et al.*, 2015). Effluent from the digester flowed through a countercurrent heat exchanger and then into a biogas-powered heater that supplied the necessary thermal energy for pasteurization. Key laboratory findings were that anaerobic digestion of undiluted human waste simulant could yield up to 0.37 NL_biogas_/g_COD_ (or 35–40 L_biogas_/p/d) in an unmixed digester at 30°C. Pathogen inactivation tests (Colón *et al.*, 2012) conducted in the laboratory using a custom-designed, full-scale biogas-powered heater/heat exchanger system sized for 10 people with an average retention time of 13 h found seven or more log reductions of *Escherichia coli* at temperatures greater than 65°C. The biogas usage for these tests ranged from 230 to 280 L_biogas_/d, only 65–75% of the expected biogas production from 10 people. From these studies, the advantage of the ADPL system is that it enables pathogen removal without relying on environmental conditions such as soil absorption and sun exposure, external power, or direct waste handling. Furthermore, the process temperature can easily be monitored for quality control purposes.

In this article, the field implementation of the ADPL concept is described. The objective of this study was to demonstrate the proof of concept in the field, show that anaerobic digestion of minimally diluted fecal waste yields enough biogas to power the inactivation of fecal pathogens by pasteurization, and provide early validation of the ADPL concept.

## Experimental Protocols

### Study site

The study took place in a peri-urban area outside of Eldoret, Kenya, known as Sogomo Estate. Typical plots in the community were characterized by 500 m^2^ of land shared by an average of 20 residents housed in rudimentary one bedroom units (Supplementary Figs. S1 and S2). Before the study, shared ventilated improved pit (VIP) latrines with concrete slabs were used on the sites. The water table ranged from 2 to 6 m below surface and was susceptible to fecal contamination by pit latrine leachate.

**FIG. 1.**
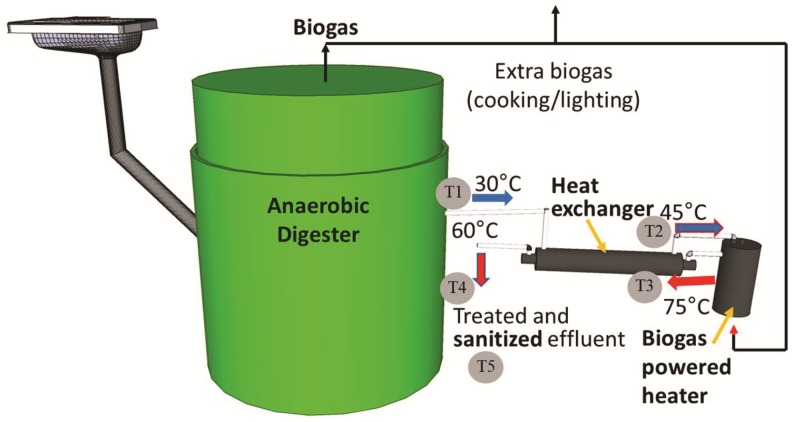
Schematic of ADPL concept with sample point Nos. 1–5. Temperature values shown are examples. ADPL, Anaerobic Digestion Pasteurization Latrine.

Two plots were chosen for the pilot study based on owner interest and having sufficient space and occupancy (Supplementary Figs. S1 and S2 for details on each plot). These systems will be referred to as Central and North henceforth. Typical occupancies of these plots over the study period were 35 and 17 residents, respectively. Residents at Central were primarily students at the nearby university and occupancy decreased over winter and summer breaks, but rarely for more than 2–4 weeks at a time. The lowest reported occupancy was 10 during both August 2014 and December 2015, but other recordings were 18 or greater. Residents at North were primarily families, and decreases in occupancy were only seen during Christmas and New Year holidays, but generally records remained between 15 and 19 residents. ADPL systems were built and used in replacement of the VIP latrines. User preferences and current sanitation habits were gathered through community surveys and were considered before system design (Duke IRB approval No. B0707). Each ADPL includes three toilet stalls connected to one anaerobic digester and a biogas-powered pasteurization system (Fig. 1 and Supplementary Fig. S3). Toilets were built above the digester to utilize gravity-induced flow. Plastic prefabricated latrine slabs (squat pan style as preferred by residents) were installed. The system did not include urine diversion. A water drum and 1 L container were provided which residents were instructed to use as a pour flush after defecating to transport feces and not to flush after urination. The change in volume of water in the storage tank for flushing was recorded and was found to be greater at North than at Central, although number of users at Central was higher indicating different personal habits. Residents were also instructed to not use cleaning chemicals in the toilets or add solid wastes, such as feminine hygiene products, that could disrupt the system's processes. Waste bins were provided in each toilet stall to minimize this risk.

### Materials and methods

ADPL schematic can be seen in Fig. 1 and Supplementary Fig. S3. The treatment systems are fully enclosed and autonomous with a 3 × 3 m footprint. Kenyan-manufactured plastic floating dome cylindrical anaerobic digesters (Gesi Safi 2000; SimGas, Nairobi, Kenya) were installed partially below grade to minimize overall height. Digestate outlet height varied slightly at each site resulting in anaerobic digester working volumes for Central and North of 2.67 and 2.52 m^3^, respectively. Assuming a daily waste flow of 2.4 L/p/d (1 L urine, 0.4 L feces, and 1 L flush) (Colón *et al.*, 2015), the estimated hydraulic residence times (HRT) of these systems were 32 and 58 days. Biogas in all digesters was held at a pressure of 3.7 cm water column using weights on top of the floating dome. Digesters were operated without mixing or external heating. Average ambient temperatures in Eldoret range from 12°C to 22°C. All flows were gravity driven, and no external energy was supplied to the ADPL system for its operation.

Anaerobic digesters were inoculated initially using effluent (about 150 L each) from a nearby dairy farm anaerobic digester. This inoculum was conditioned by adding primary residential sewage (3 L/d) over a 2-week period to acclimate to human waste before adding the inoculum to the installed anaerobic digesters. Once inoculated, the digesters were fed gradually increasing quantities of organics (cow manure 1–8 kg_wet_/d) and nitrogen (urea fertilizer 0–90 g-N/d) over 4 weeks before opening systems to full usage by residents. The purpose of this startup procedure was to prevent organic overloading at start and acclimate methanogens to high ammonia concentrations to minimize ammonia inhibition, as was demonstrated in Colón *et al.* (2015).

Effluent overflow from the digester passed through a heating system composed of a counter-current tube-in-shell heat exchanger (1.9 L tube, 7.8 L shell) and heater (7.7 L) (Supplementary Fig. S3). The lowest average HRT in the heater based on daily usage pattern estimates would take place at Central, 2.2 h, that is, well-above the time threshold of 10–15 min for pathogen inactivation at 70°C mentioned above. These heater/heat exchanger units were fabricated by a local welder using sheets of 16-gauge galvanized steel following designs developed by our team. Heat for pasteurization was provided by burning biogas with a Bunsen burner below the heater. The biogas flow was kept constant (200 mL/min) using a simple RMA-13 Dwyer rotameter (Michigan City, IN). The pasteurized final effluent from the heating system flowed into a 200 L drum. The drum was installed for temporary effluent storage. It currently overflows into existing pit latrines, although the vision is to use the sanitized effluent as a fertilizer in the future.

Installation was completed, and toilet usage began in August 2013. However, reliable monitoring activities were not able to be initiated until April 2014.

Two ADPL systems were monitored daily for biogas production, heating system temperature, and general operation. Biogas production was calculated by measuring the change in dome height between daily readings and adding the daily gas flow into the Bunsen burner. Temperatures were taken using a handheld digital thermometer at the inlet to the cold chamber of the heat exchanger (T1), the outlet of the cold chamber (T2), inside the heater (T3), the outlet of the hot chamber (T4), and the effluent collection container (T5). After October 2015, Arduino microcontrollers were installed with thermistor temperature probes for continuous monitoring of the heating system at points 1–4.

Grab samples were periodically taken and analyzed at ELDOWAS (Eldoret municipal water and wastewater treatment center) for the following parameters at the digester outlet: BOD_5_ (Standard Methods 5210 B), chemical oxygen demand (COD; Hach Company), total suspended solids (TSS; Standard Methods 2540 D), pH (Standard Methods 4500-H^+^ B), and total ammonia nitrogen (TAN; Hach Company). Sampling of the inlet was attempted; however, collecting representative samples was not possible due to the heterogeneous nature of fresh fecal sludge and its discontinuous flow. In addition, ELDOWAS performed tests to determine FC counts on samples taken from heating system point Nos. 1–5 using the Colilert method.

## Results

### Operation, biogas production, and temperatures in the pasteurization systems

Adoption of the two ADPL systems was successful, and residents reported they preferred the ADPL to their pit latrines, due to the ADPL having little to no odor or flies. Usage of the toilets was high, and the residents generally complied with the instructions provided. The use of flush water was assessed by monitoring the daily change in volume of water in the storage tank for flushing. Flush water usage was found to be greater at North than at Central despite the higher occupancy at Central. Thus it is believed that residents at Central did not use flush water after each defecation as instructed. Operators occasionally found thick solids causing clogs in piping from the toilets to the digester. This was more common at Central than at North, which supports the assumption of lower flush water use at that location.

**FIG. 2.**
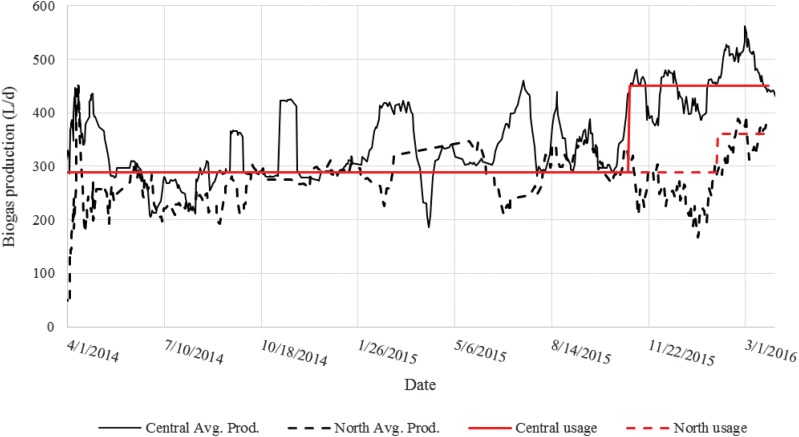
Ten-day average biogas production rates for Central and North and average biogas usage in the heating system.

Biogas production data are reported in Fig. 2. Although relatively steady operation of the anaerobic digesters was achieved during the entire reporting period (with some seasonal variations), individual measurements of biogas production exhibited a large amount of scattering. This was caused by the method of calculation and will be discussed below. Average biogas production over the total study period from Central and North was 409 and 273 L_biogas_/d, respectively. Assuming a waste loading of 100 g_COD_/p/d and the typical occupancies of 35 and 17 at Central and North, the estimated biogas yield was 0.12 and 0.16 m^3^_biogas_/kg_COD_. This biogas yield is lower than the median of 0.20–0.25 found in previous studies (Lettinga *et al.*, 1993; Kujawa-Roeleveld *et al.*, 2003; Chaggu *et al.*, 2007; Mang and Li, 2010; Tilmans *et al.*, 2014) and 0.37 m^3^_biogas_/kg_COD_ in the laboratory study (Colón *et al.*, 2015). Several explanations exist for the alleged lower biogas yield. First, toilet usage and input COD are rough estimates (maybe only accurate ±20% or 30%) and, as mentioned before, quite difficult to determine with greater accuracy. Second, biogas production values are potentially underestimated because of the method of calculation by gas usage plus change in collection volume. During periods in which biogas production was greater than usage, the excess gas volume would at times exceed that of the collection dome and escape from the sides. This scenario occurred on 8% and 29% of total monitoring days at North and Central, respectively. This volume could not be quantified and was not included in the biogas production rates. The impact of the loss of excess gas in calculation of production rate was seen at Central when biogas flow to the Bunsen burner was increased from 288 L_biogas_/d to an average of 450 L_biogas_/d from November 2015 to March 2016. The calculated average biogas production rate resulting from this change in burner use increased from 350 L_biogas_/d to 461 L_biogas_/d (Fig. 2). Increased gas flow to heating system was tested at both sites during February 2016, which resulted in being the month with the highest average gas production for both locations, 509 and 342 L_biogas_ produced per day at Central and North, respectively. The corresponding yield during this month was 0.16 and 0.18 m^3^_biogas_/kg_COD_, which is closer to, but still less than the expected range.

**FIG. 3.**
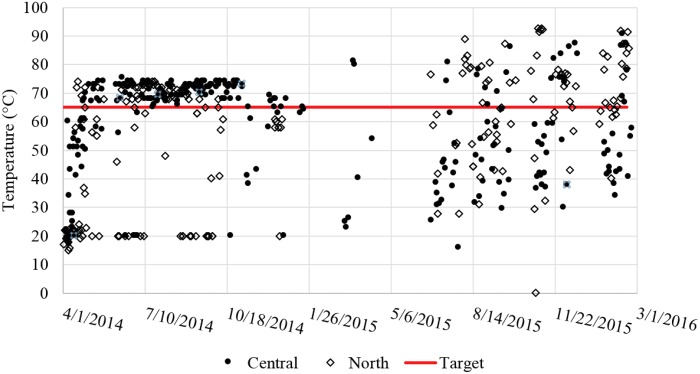
Daily temperature measurements at Central and North with target minimum temperature.

Biogas flow to the Bunsen burner was set to a constant value (i.e., always on) and was initially adjusted to not consume more gas than produced on average while still maintaining the heating tank temperature in the proper range (65–75°C). The best flow was found to be 200 mL/min (288 L/d), which was used at both sites for the majority of the study. Note that this value is expected to be site and system specific. Assuming a biogas composition with 60% methane, this flow corresponds to 70 watts of calorific energy input to the heating tank. The most stable period of operation was May to December 2014 as seen in Fig. 3. The temperature in the heating tank was measured to be >65°C for 87% of measurements (*N* = 137) at Central and for 58% of measurements at North during this period. ADPLs at both locations used an average biogas flow of 288 L/d during this period. Monitoring activities from January to June 2015 were limited due to reduced local student involvement and issues with monitoring devices. From June 2015 to March 2016, biogas flow to the burner at Central was increased to an average of 380 L_biogas_/d while the flow at North remained at 288 L_biogas_/d until February 2016, after which flow was increased to 360 L/d. During this period, heating performance at Central declined and reached >65°C on only 28% of sampling days (*N* = 183), even with higher gas flow. Performance at North remained the same at 59% of sampling temperatures >65°C.

Hardware failures, accumulation of solids in either the heater or the heat exchanger, inconsistent waste flows and blockage of flow by large solids, and occasional low biogas supply were identified as some of the possible reasons for failure to reach target temperatures. Over the full study period, the flame was discovered to be off for 15% and 38% of the total number of days of monitoring (*N* = 407) at Central and North, respectively. Sufficient biogas was flowing during 71% and 53%, respectively, of the occurrences in which the flame was found to be off, meaning burner hardware issues were the primary cause for the flame to go out. While considering the amount of days the target temperature was not reached, these hardware issues account for 14% and 34% of those days. The Bunsen burners used were susceptible to corrosion (moist biogas containing H_2_S) and clogging, making it difficult at times to maintain the flame. Wind was an additional cause for the flame to go out, so wind barriers were installed and appeared to be effective in preventing wind from extinguishing the flame. Biogas H_2_S scrubbers consisting of a 60 cm long 5 cm ID PVC pipe packed loosely with household steel wool were installed between the dome and the gas flowmeter to remove H_2_S. Still, the Bunsen burners were subject to corrosion, which was believed to be due to residual H_2_S and the natural humidity in the biogas.

**FIG. 4.**
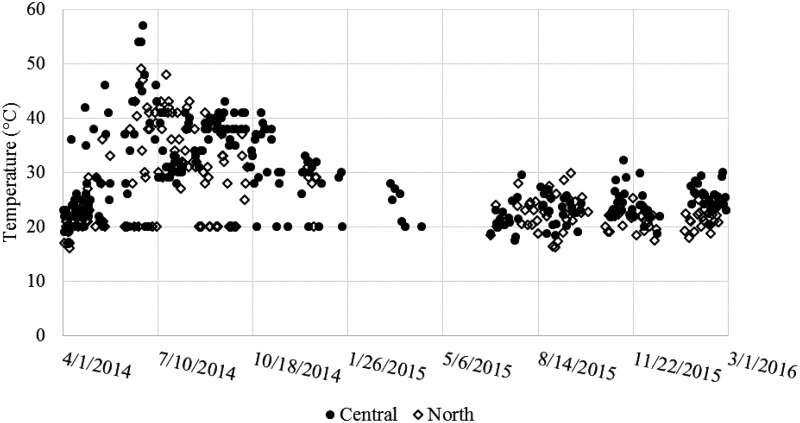
Daily temperature measurements from outlet of cold chamber of heat exchanger (point No. 2). This point is also the inlet of the heater.

During the June 2015 to March 2016 period, a greater number of blockages in the digester outlet and heating system were observed together with an accumulation of solids in the heating system. These issues are all believed to be due to an increased amount of solids leaving the digester after 2.5 years of operation without desludging. Accumulated solids settling in the heater and in the heat exchanger were most probably reducing heat transfer due to lower liquid residence times and fouling of surfaces. These effects were not quantified, but their occurrence correlates with the inconsistent temperature measurements seen in Fig. 3, as well as decreasing trend of temperatures at the cold effluent port (point T2) shown in Fig. 4.

Arduino microcontrollers were installed in January 2016 to provide continuous temperature readings of the system. These readings highlight challenges associated with variable usage of the latrine and flow blockages. As mentioned, the biogas flow was held constant, but the highly variable waste flow during a day (high toilet usage early morning, no usage during nighttime, and occasional clogs blocking flow) meant that each day includes significant temperature fluctuations: high temperatures during low flow times and low temperatures after high toilet usage. Blockages in the digester outlet pipe and heat exchanger inlet also caused instances of variable flow as treated effluent would backup into the digester before a large pulse would flush through the pasteurization system when the blockage was cleared.

**FIG. 5.**
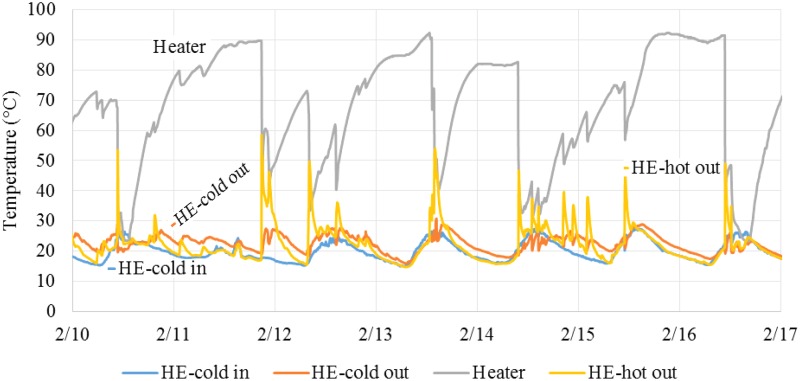
Temperature readings at Central during a week in February 2016. Points monitored include (T1) Heat exchanger cold chamber inlet, (T2) Heat exchanger cold chamber outlet = heater inlet, (T3) Heater, and (T4) Heat exchanger hot chamber outlet.

Figure 5 shows the details of 1 week of operation during February 2016 at Central. The average temperature over this period was 67.5°C, providing evidence that the energy supply was sufficient to pasteurize the overall waste flow; however, only 60% of the discrete temperature measurements were >65°C. Large drops in heater temperature were seen midday on 2/10, 13, 14, and 16. These are times at which the operator visited the system and cleared a blockage. Heater temperature lines are relatively smooth before these times, indicating that little effluent flowed through the system. Another large drop in temperature was seen on 2/11 near midnight. This time was later than the operator's visit and assumedly a low usage time as most residents would not have been active. This drop is most likely in response to a blockage cleared due to pressure buildup in the system. Another large drop was seen on 2/12 at 7:45 am. This drop is assumed to be from a peak usage period causing high flow through the system. Smaller drops in heater temperature were seen on 2/12 and 14–15. These were expected based on normal toilet usage.

### Chemical and biological analysis of ADPL effluent

Table 1 shows the average values for COD, biochemical oxygen demand (BOD), TSS, pH, and TAN for the ADPL effluent at North and Central locations for sampling in June to July 2015 and February to March 2016. As mentioned in the Materials and Methods section, representative sampling of the digester influent was not possible because of its heterogeneous nature, and thus, one had to rely on assumed values. Jönsson *et al.* (2005) found a global average COD concentration of 72,100 mg_COD_/L for fresh human excreta. Assuming excreta (400 g_feces_ and 1 L_urine_/p/d) was diluted with 1 L of flush water per person per day, the influent COD in our study was about 42,100 mg_COD_/L. Based on this assumed value, COD removal efficiencies at North and Central were 89% and 85%, respectively. The higher effluent COD concentrations observed at Central were most likely due to the higher loading rate and shorter retention time at that site. The pH remained within a suitable range for anaerobic digestion (average of 7.4 and 7.7 at North and Central, respectively). The concentrations of TAN (2,420 and 4,750 mg NH_3_-N/L at North and Central, respectively) showed that the effluent was nutrient-rich, which is positive, given the intended reuse locally of the pasteurized effluent as a fertilizer. Monitoring of the flush water tank revealed that residents at Central used less flush water than at North. The higher total loading and decreased water usage at Central resulted in a more concentrated waste loading, which likely explains the higher TAN concentration at Central. Differences in the use of the ADPL for urinating (urine contains 80–90% of the 7–10 g nitrogen excreted per person per day; Colón *et al.*, 2015) could also exist, but may not be as important as the differences in flush water usage.

**Table 1.** Average Values and Standard Deviation for Results of Anaerobic Digestion Pasteurization Latrine Effluent

	*North*		*Central*	
	*Avg.*	*SD*	*Avg.*	*SD*
COD (mg/L)	4,540	2,550	6,450	3,530
BOD (mg/L)	2,050	1,308	3,970	1,990
TSS (mg/L)	2,130	1,620	3,570	2,200
pH	7.4	0.7	7.7	1.0
TAN (mg-N/L)	2,420	506	4,760	1,090

*N* = 6, sampling during June to July 2015 and February to March 2016.

BOD, biochemical oxygen demand; COD, chemical oxygen demand; SD, standard deviation; TAN, total ammonia nitrogen; TSS, total suspended solids.

**FIG. 6.**
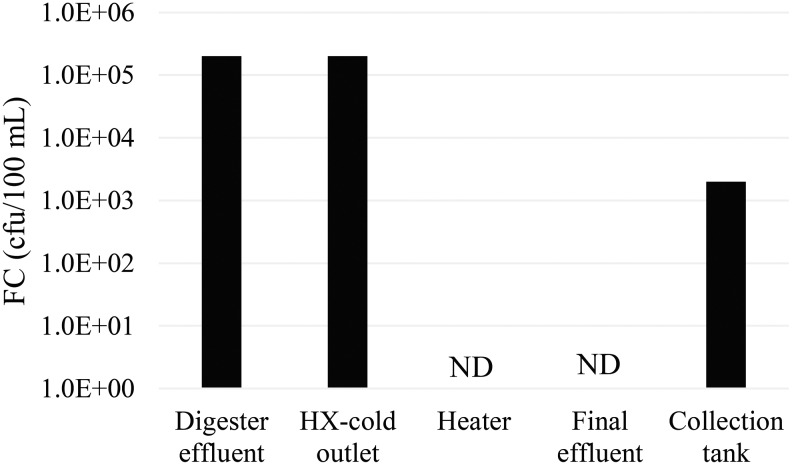
Fecal coliform (FC) concentrations measured in North's pasteurization system (February 2016 sampling). HX, heat exchanger.

FC was used as the indicator for pathogen inactivation in limited sampling. Samples (*N* = 3, sampled in February 2016) were taken on days during which the heater was measured at >65°C and results are shown in Fig. 6. Readings before the heater exceeded the laboratory's upper detection limit of 201,000 colony forming units (cfu) per 100 mL, while no detectable colonies were in the heater on each of these days (lower limit of detection was 1 cfu per 100 mL). Thus >5 log reductions of FC were achieved in the pasteurization system of the ADPL, as expected from time-temperature charts for various pathogens reported by Feachem *et al.* (1981). FC concentrations in the outlet of the heat exchanger cold chamber and in the final collection tank varied with time and operation. Little or no disinfection occurred on the cold side of the heat exchanger because temperatures were too low. The concentration of FC in the collection tank exceeded 1,000 cfu per 100 mL (Fig. 6); this was most probably not caused by regrowth, but rather due to carry over of unpasteurized effluent from instances during which temperature in the system was below the 65°C target. Obviously, more extensive monitoring of pathogens is needed, including more difficult to inactivate pathogens, such as helminths.

## Discussion

The ADPL concept was successfully proven in the field in Eldoret, Kenya. The biogas yield over the period of evaluation was lower than expected, but the value is subject to a significant (yet unknown) uncertainty. The low calculated yield could be due to several factors resulting from the limitations of field studies. Homogenous sample of the digesters' influent was not obtained, and influent characteristics were therefore assumed based on literature data. Influent waste and treated effluent flows were also not accurately known, due to limitations in the field, and were based on the assumptions that all residents used the ADPL and that waste volumes per resident equaled values from literature. In addition, as mentioned in the Results section, there is evidence that not all biogas that was produced was accounted for, and thus, biogas production values are underestimated. Biogas yield values are, therefore, best estimates and further efforts should be directed toward reducing the uncertainty on its value.

Target temperatures were reached for large portions of the study (87% and 58% of the time, at Central and North, respectively, for May to December 2014, or 62% and 61%, respectively, for the entire study period) powered by the biogas produced by the anaerobic digester. Many of the occurrences of heater temperature being too low were not caused by a lack of biogas supply. One example is seen by comparing Central from 2014 to 2016 (Fig. 3). The temperature measurements were >65°C for 87% of the readings in 2014. This percentage dropped to 24% even though the average biogas flow to the burner was increased from 288 to 380 L_biogas_/d. This example points to a significant decrease in thermal efficiency and increase of hardware issues instead of insufficient biogas supply. The decrease in heat exchange efficiency over time is seen in Fig. 4 and also contributes to the decreased heating performance.

Variable toilet usage (and thus waste flow) during the day represented another challenge for maintaining pasteurization temperatures. Periods with little to no flow resulted in temperatures higher than the desired range and, thus, wasted biogas. In contrast, peak toilet usage periods often caused the temperatures to decrease below the target, with the added issue of reducing the retention time of the treated effluent in the pasteurization system. These effects can be seen in Fig. 5 where the average temperature (67.5°C) was in the desired temperature range, but many incursions below 65°C were observed. This resulted in a large portion of the total volume that was not adequately treated. These findings provide motivation to regulate either waste or biogas flow, so that either the liquid flow is equalized throughout the day or that biogas supply is matched to the heating needs of the pasteurization system. Inexpensive microcontrollers and simple hardware (gas valve and burner igniter) have significant potential to resolve this issue and are currently being exploited by our team.

Based on the analysis of these results, the concept that the energy generated by anaerobic digestion of human waste is sufficient to power its own pasteurization was demonstrated. The biogas produced was able to heat digester effluent to the target temperature range of 65–75°C. No detectable fecal colonies were found at sampling occurrences when the heater temperature was >65°C. These results confirm that pasteurization is an effective pathogen control method. However, the finding of FC in the effluent collection tank highlights the need for maintaining >65°C at all times to ensure safe effluent reuse or disposal. Improvements in system design to increase biogas yields, decrease clogging, improve thermal efficiency, decrease operation and maintenance needs, and match energy and waste flows will improve performance and make operation more reliable. In addition to ensuring that treatment goals are met, these improvements will also improve the likelihood of having excess biogas for use in other applications and providing a safe effluent for reuse as fertilizer.

ADPL systems are relatively simple to build and maintain, and all materials for the construction and installation were purchased or fabricated locally. ADPLs do not require energy input and, thus, can be operated off the grid. Further economic study is needed to determine if the ADPL is a cost-effective sanitation alternative, but a preliminary assessment found the total cost of the initial installation and material upkeep was roughly $2,000–3,000 per system, for 20–30 users. With a 10-year life for the digester and replacement of the heat sterilization system every 2–5 years, the overall cost comes to about 1.8 to 4.1 cents per user per day (without factoring energy/fertilizer benefits), which is within an appropriate range (<5 cents per user per day) as has been suggested elsewhere (BMGF, 2011).

## Conclusion

Two full-scale ADPL systems were implemented and tested in field conditions. Results from this study showed that anaerobic digestion of minimally diluted fecal sludge can yield sufficient energy in the form of biogas to power pasteurization of its treated effluent. This demonstrates that pathogens in human excreta can be removed by exploiting the wastes' native properties and without relying on external energy or environmental resources. Target temperature goals were not always met in this study, resulting in some digester effluent that was inadequately treated. This failure, however, was mostly due to operational issues, rather than biogas shortage, indicating that ADPL systems need improvements in consistency and reliability. Overall, this study suggests that the ADPL system could be a feasible alternative for on-site sanitation by providing effective control of fecal pathogens before effluent reuse.

## Supplementary Material

Supplemental data
