# Supplementary material for: On-Site Fecal Sludge Treatment with the Anaerobic Digestion Pasteurization Latrine
Source: Environ Eng Sci. 2016 Nov 1;33(11):898–906. doi: 10.1089/ees.2016.0148 (PMC5134843; doi:10.1089/ees.2016.0148)
Supplement: Supplemental data [file Supp_Data.pdf]

Supplementary Information

Onsite fecal sludge treatment with the Anaerobic Digestion Pasteurization Latrine

Aaron A. Forbis-Stokes, Patrick F. O'Meara, Wangare Mugo, Gelas M. Simiyu, Marc A. Deshusses[1]
[1]Department of Civil & Environmental Engineering & Duke Global Health Institute, Duke University, 127C Hudson Hall, Box 90287, Durham, NC 27708. *Phone:* (919) 660-5480; *Fax*: (919) 660-5219.*; emails:* marc.deshusses@duke.edu, aaron.forbis-stokes@duke.edu

North

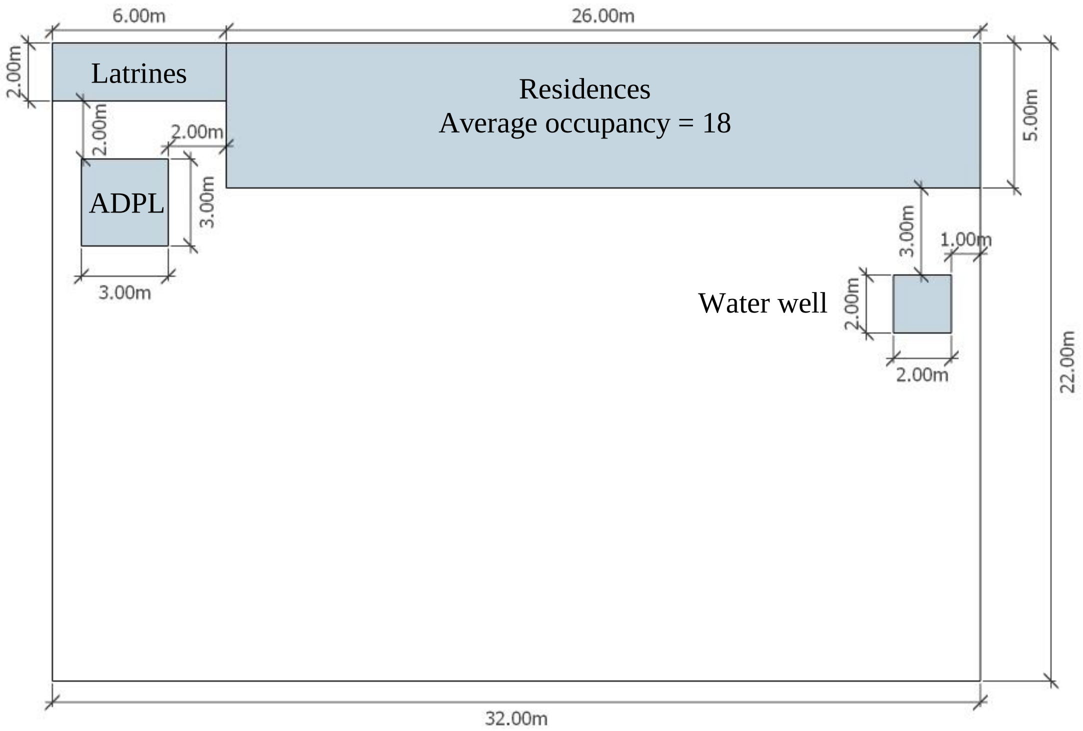

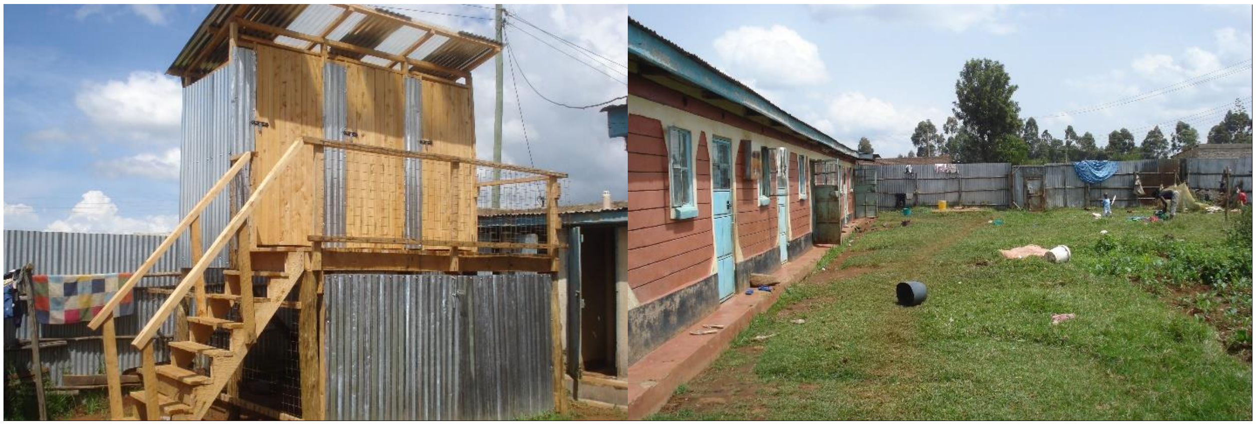

Figure S1. Layout of North (top), view of ADPL located next to existing latrines (bottom, left), and the view of the plot from the ADPL (bottom, right).

Central

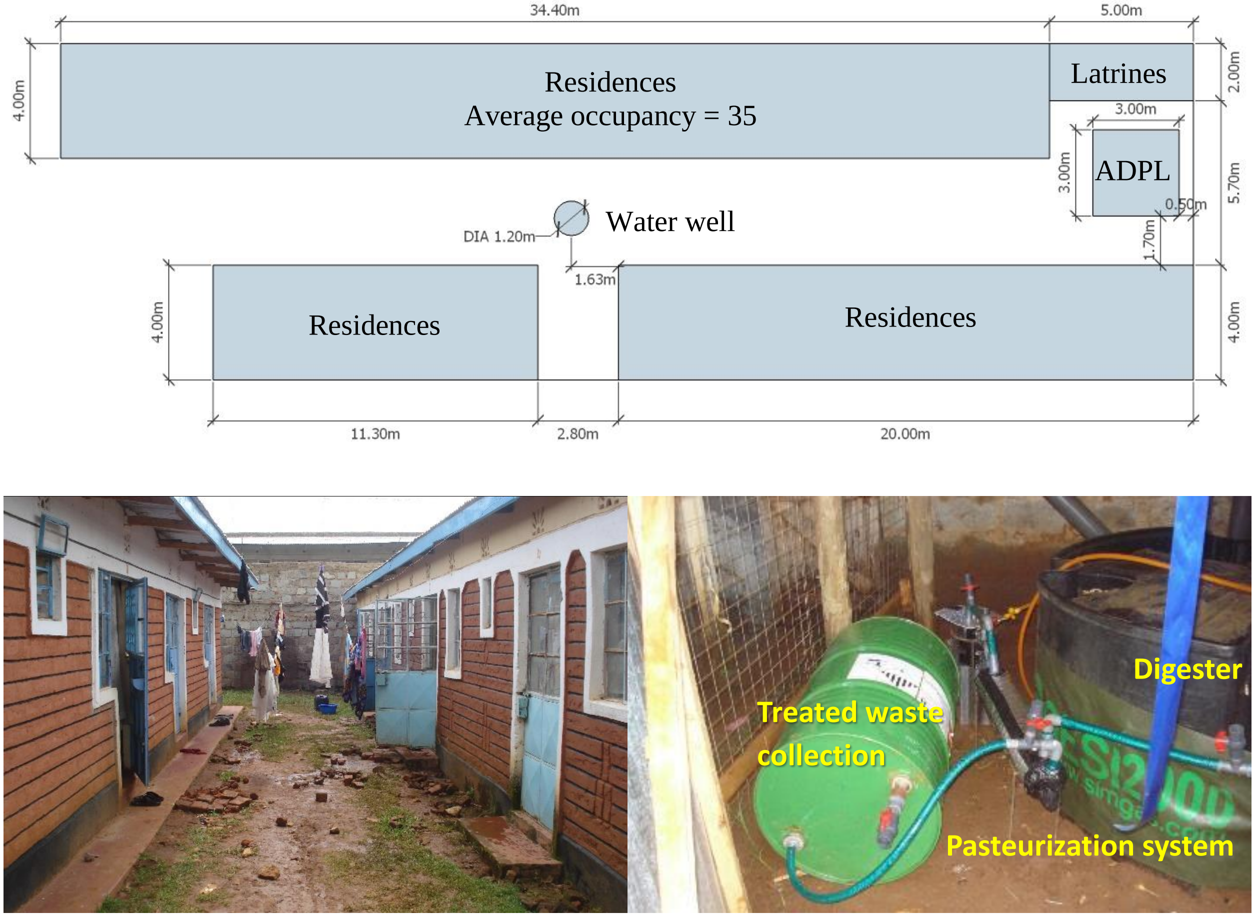

Figure S2. Layout of Central (top), view of the plot looking towards the location the ADPL was installed (bottom, left) and view of the treatment system enclosed below the latrines superstructure (bottom, right).

Heating system

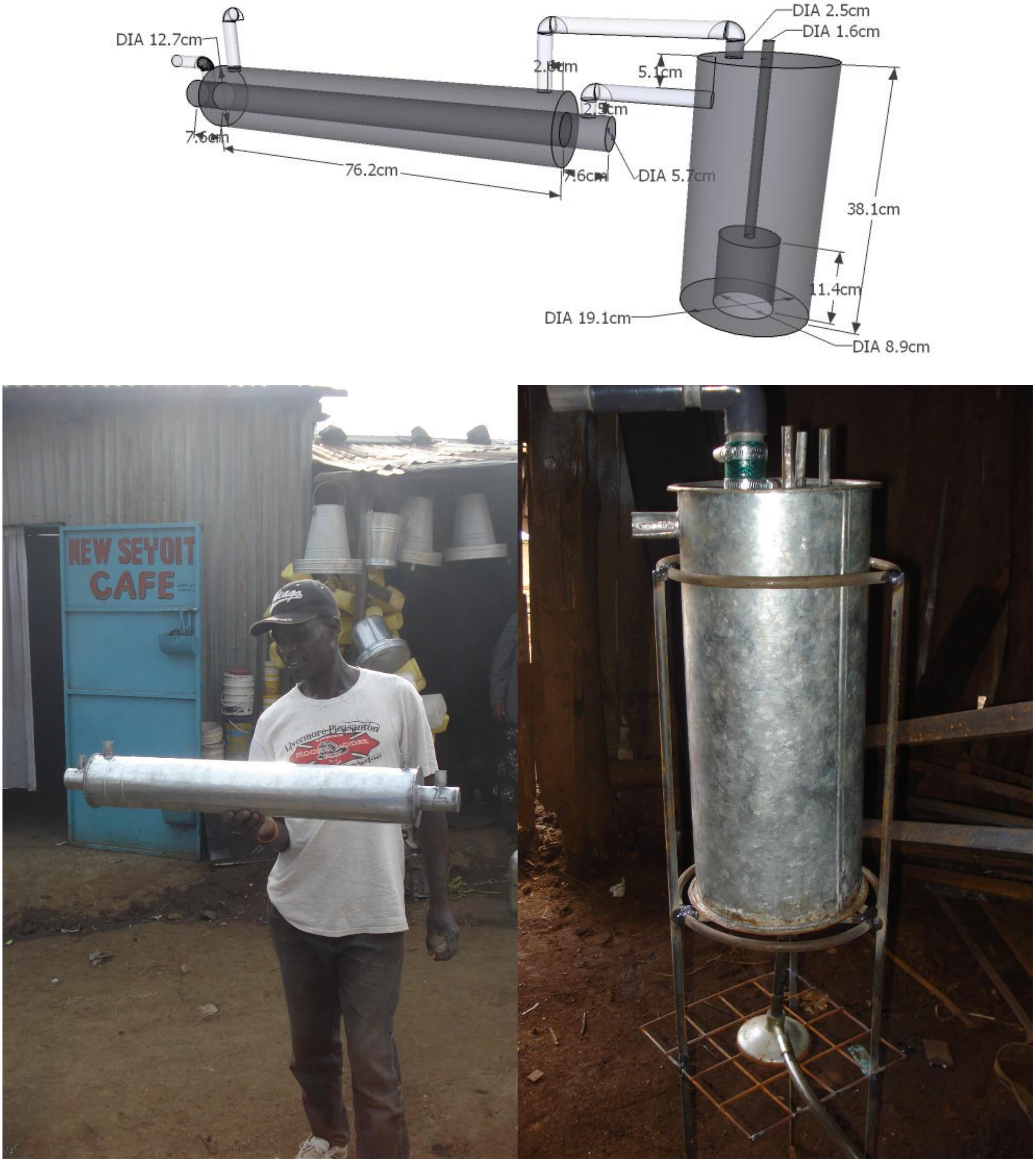

Figure S3. Schematic of heating system (top) and locally fabricated heat exchanger (bottom, left) and heater (bottom, right).
